# The Nursing Supplement

**Published:** 1890-03-08

**Authors:** 


### "The Hospital" Nursing Mirror.

Being the Extra Nursing Supplement of "The Hospital" Newspaper.

All Contributions for this Supplement should be addressed to the Editor, The Hospital, 140, Strand, London, W.C., and should have the word "Nursing" plainly written in left-hand top corner of the envelope.

### En Passant.

Exhibition of Nursery Uniforms.—The doll show at Shrewsbury was much enjoyed by all. One of the accident wards, the use of which was kindly granted by the directors, was beautifully decorated with snowdrops, ferns, and palms, and a crimson stand was erected to hold the dolls. The nurse dolls, when unpacked, had their caps put straight and their hair brushed, and looked quite trim and tidy, and none the worse for their travels. The visitors were numerous, and the skill and handiwork of the dressers of the dolls was much admired.

Short Items.—The *Women's Penny Paper* has started a "Nurse's Column." Is this the work of the new sub-editor?—Mrs. Marsden, of Manchester, is trying to provide amusement for hospital nurses; twice she has entertained large parties of nurses at tea.—"Miranda," writing in the *Ladies' Pictorial*, asks why nurses wear useless veils, which are distinctly in the way on a windy day.—Midwives in Prussia are bound by law to follow certain antiseptic precautions, in which the nail-brush largely figures.—In Paris midwives are recommended to use as an antiseptic a mixture of 50 centigrammes of tartaric acid and 25 centigrammes of sublimate, with some colouring matter.

The Glasgow Training Home.—The annual meeting of this old-established and excellent home was held last week. Mr. Forbes, secretary, read the sixteenth annual report of the directors, which bore that during the past year 172 patients had been treated in the home, and of these 124 were surgical cases. Trained nurses had been sent out to wait on 483 cases of sickness in private families, which showed an increase of 14 over the previous year. Mr. Robert Gourlay, treasurer, submitted a statement of accounts for the financial year ending December 31st last, from which it appeared that the income, including a balance of £51 5*s.* from the previous year, amounted to £4,554 1*s.* 6*d.*, and that the year closed with a balance on hand of £535 12*s.* 8*d.*, which the directors had meantime transferred to the building fund account. Further accommodation for the 79 nurses attached to the home is badly needed, but there is yet a debt of £2,000 on the home.

Thousands of Pounds for the National Pension Fund.—We have the pleasure to acknowledge the following sums towards the £14,000 now being raised to at once bring the Donation Bonus Fund up to £40,000. We hope to be able to acknowledge contributions amounting to £1,000 every week until the £40,000 are completed. Contributions of any amount may be sent to the Editor of The Hospital, or to the Manager, National Pension Fund for Nurses, 8, King Street, Cheapside, London, E.C.:

The Seventh Thousand.

| | | | |
|---|---|---|---|
| Messrs. Brown, Shipley & Co. ... ... | £525 | 0 | 0 |
| Messrs. Hichens, Harrison & Co. ... ... | 100 | 0 | 0 |
| Messrs. Mullens, Marshall & Co. ... ... ... | 100 | 0 | 0 |
| Mr. J. H. Renton... ... | 100 | 0 | 0 |
| Messrs. J. K. Gilliatt & Co. | 50 | 0 | 0 |
| Mr. W. C. Alexander ... | 20 | 0 | 0 |
| Messrs. Harris & Co. ... | 10 | 10 | 0 |
| Salters' Company ... | 10 | 10 | 0 |
| Mr. A. Spicer ... ... | 10 | 10 | 0 |
| Mr. W. C. Burns... ... | 10 | 0 | 0 |
| Mrs. W. Burns ... ... | 10 | 0 | 0 |
| Mr. Fred Cox ... ... | 10 | 0 | 0 |
| Mr. W. Brander ... ... | 5 | 5 | 0 |
| Mr. Sydney Lawrence ... | 5 | 5 | 0 |
| Mr. A. Symes ... ... | 5 | 0 | 0 |
| Mr. Gordon Miller ... | £5 | 0 | 0 |
| Mr. C. Wood ... ... | 2 | 2 | 0 |
| Mr. W. White ... ... | 2 | 2 | 0 |
| Messrs. J. P. Stearns & Co. | 2 | 2 | 0 |
| Mr. Clifford Wigram ... | 2 | 2 | 0 |
| Mr. J. Hall... ... ... | 2 | 2 | 0 |
| Mr. F. C. Carr Gomm ... | 2 | 2 | 0 |
| Mr. W. Capel Slaughter | 2 | 2 | 0 |
| Nottingham & Notts. Nursing Association ... | 2 | 2 | 0 |
| Mr. J. Yule ... ... | 1 | 1 | 0 |
| Mr. W. J. Phillips ... | 1 | 1 | 0 |
| Mr. C. Baudin ... ... | 1 | 0 | 0 |
| Messrs. R. B. Marzetti & Co. ... ... ... | 1 | 0 | 0 |
| Mrs. Sandbach ... ... | 1 | 0 | 0 |
| Mr. A. Bainbrigge ... | 1 | 0 | 0 |

Amount already acknowledged **Seven Thousand Pounds.**

Cheltenham District Nurses.—The report of last year's work is a good one. The staff now consists of a resident lady superintendent (L.O.S.), two midwives (L.O.S.) and two trained nurses. The income last year was £637, and of this £100 has been set aside as a reserve fund. At the annual meeting Miss J. Wilson delivered an interesting address in favour of full training for nurses.

Registration of Midwives.—At the annual meeting of the Obstetrical Society, the President said that the number of women who presented themselves for examination was largely increasing. The society was in favour of legislation to establish a register for midwives; the midwives to be in no way connected with the proposed voluntary register for nurses. The society was, therefore, glad to see that Mr. Fell Pease had introduced a Bill into Parliament for the compulsory registration of midwives. If any of our readers have taken up this branch of their profession, and are not yet members of the Midwives' Institute, we advise them to write at once for particulars to the secretary, Mrs. Nicholl, 12, Buckingham Street, Strand, W.C.

Worcester News.—The Worcester City and County Nursing Institution has ended the last year with a debt of £104. Nine private patients were nursed in the home during the year, and a total staff of 37 nurses and seven probationers were kept at work either amongst the poor or in private families. The district nurses at Worcester wear very becoming crimson stuff dresses, and the usual black cloak and veil. The St. John's House nurses are distinguished by white strings to their bonnets and the absence of the veil. The matron of Worcester Infirmary, Miss McLelland, has just had her efficient management approved in a practical manner by a rise in her salary; everyone is rejoiced, as under her rule the infirmary nurses are certainly better trained, and all things work peacefully and well. The lectures given by the house-surgeon are highly approved of by the infirmary nurses, and are really excellent. Worcester quite feels now, that in nursing matters, it keeps pace with the times.

Nursing at Berwick.—A letter to the *Berwick Journal* takes exception at the inadequate nursing staff of Berwick Infirmary. A short time ago, at Berwick, four men were seriously burnt, one hopelessly so. "The burns were dressed by the doctors, who left the one matron to watch by the dying man, and to look after the other three. The two men who were burnt would want constant attention, and who was there to give it? The young man died at midnight. The servant who ought to have been in bed to be ready for the next day's work was the only assistant left for the matron. What ought to have been done for that young man would take at least two hours. Who was looking after the others, and what must they have felt at the sight? The matron was obliged to send for a man to help her to carry the body away. Then followed the long watching by the others, neither the matron nor servant getting any rest. Why should they have been left at any time to the tender mercies of an inexperienced servant girl? The matron got no proper help, until, worn out and depressed, she broke down five days after the accident, when a nurse was sent for, and had to take charge of the matron, who was really very ill, and of the two now dying men. The first night after her arrival, one man was delirious and died, the other needing constant attention, and the matron also. When was the nurse's time for rest? On the following week a little child was brought in, and as there was no one to look after him, the mother had to take him home again."

### The Story of a Sermon.

It was afternoon—the still, drowsy afternoon of a perfect summer Sunday. Seated in comfortably cushioned wicker-chairs, under a walnut tree in their old-fashioned garden, the two Miss Usherwoods were contentedly drinking in the beauty of the distant "landscape winking through the heat." On their laps lay good books, for the Miss Usherwoods were very orthodox—in truth, they were simple-hearted folk, and without guile—but the books were unopened; it seemed enough to glance languidly, now and again, at their smart covers.

"Sister"—it was Miss Selina, the youngest, who, at length, broke the long pause—"what did you think of this morning's sermon?" The question was put nervously, for there had been a constrained silence between the ladies, regarding the oration delivered, that day, in the ivy-covered village church, by a stranger, a London man.

"I was considerably impressed by it, Selina," was the reply, in measured tones, for the elder Miss Usherwood was nothing, if not elaborate in her speech.

Between herself and the other Miss Usherwood, there was a wide gap of years; one being the eldest of a large family, the other the youngest. By dint of a pathetic delusion, Miss Selina was still a maiden in her prime in Miss Usherwood's dim eyes which failed to note how the years had robbed the younger sister's cheek of its roundness and tints; her hair of its brown luxuriance; her figure of its youthful spring. But it would have been a hard heart that ventured to shatter the gentle, harmless fiction; so Miss Selina went on deferring, and Miss Usherwood guiding and controlling as if the clock of time had stopped.

"Yes; I was impressed; after the first shock," went on the elder lady leisurely fanning herself with a huge, antiquated green fan—a fan that looked as if it could have whispered of many a past-and-gone romance; "for these new people with their new ideas are somewhat startling. Certainly, this good, pious man has opened the eyes of my understanding. Selina, my child, I repeat I was impressed deeply by his relation of the poverty, the 'aching misery' as he put it of that terrible London. Ah me! Little did I dream, long years ago, when I, a thoughtless little girl visited the famous city, of the black side of what I thought a dazzling picture of gaiety and pleasure!"

In her far-off youth, Miss Usherwood had, once, spent a fortnight—an oasis of brilliant dissipation in her monotonous village life—in the Metropolis, and she had, to a large extent, lived upon the reputation of that eventful fortnight, at least, in her less fortunate sister's eyes.

"Yes, sister;" eagerly answered Miss Selina, "but think, *think* how London has doubled, trebled itself since then; and with its size, its wretchedness has increased proportionately." Miss Selina quoted—and she knew it—from the sermon, but the sentence seemed so well-rounded that she blushed with as much conscious pride as if it were original.

There was no reply and another long silence ensued.

"Sister," began Miss Selina breaking it, "I need not remind you, with your memory, but those closing remarks of the sermon, did you approve, did you agree with them?"

"You mean," and the green fan halted its undulations as as if to listen," the stern injunctions of the preacher to carry out his text: 'Bear ye one another's burdens'? I did approve, Selina, I did."

"Oh, sister, how happy I am hear you!" half sobbed Miss Selina, "I was afraid—you are so superior, you know, I dared not hope you would share my feelings. And—cannot we help to bear the burden of some poor fellow-creature, shut up to starve and die in a prison of grimy streets?" Miss Selina spoke from her heart; she had been deeply moved by the sermon preached that summer Sunday—moved to the innermost depths of her nature, depths stirred for the first time.

"Dear child!" Miss Usherwood spoke patronisingly but practically, "It is very sweet of you to have such a feeling. But what can we, with our small, inelastic income, do? Why, fortunes thrown away wholesale into the gulf of human misery would not, could not, leaven the vast lump of ———"

"Oh, but," interrupted the younger lady, "he—that clergyman—said if we each did what we could, and it is our sacred duty to do so, that it must, it would help. Now, I have an idea, sister, a poor, foolish one, you will think——"

"Let me hear it, my dear," Miss Usherwood condescended to ask.

"It is this:" Miss Selina spoke timidly; "it is true we have but a tiny income, but we have this old home of ours with its shady garden. We have many comforts—"

"Necessities" corrected the listener.

"Yes, yes;" Miss Selina waxed more nervous, and the wicker chair creaked sympathisingly. "Sister, I've been thinking, could we not go without our new velvets, and, with their cost, have down, here, for the summer months, some poor, pining city waif!"

It was out, at last, and Miss Selina sat palpitating, and fearing to raise her eyes. It was a womanly suggestion, but then, Miss Selina was an old-fashioned woman, such as our mothers used to be; not like our intellectual, highly-cultured selves, of course. The Miss Usherwoods had a practice, peculiar to themselves; they wore undeviatingly, for evening *toilette*, black velvet gown (the *velvet* was an amiable fiction, velveteen being the actual material). The ladies, methodical in all things, had stated periods when new black velvets were purchased, the superseded robes being relegated for home wear. An epoch of this sort was now approaching; thus Miss Selina's suggestion.

Another long silence; Miss Usherwood was digesting the novel idea.

"Pray, how could we get at a genuinely deserving case?" The question was curtly put, but Miss Selina's heart leaped; the case was her own.

"Why, sister, who could be better to ask than nephew Philip?" she demurely asked, and before Margery, their old retainer, brought out their cups of tea, the ladies had settled the matter.

* * * *

The Rev. Philip Usherwood was a hard-working curate in Westminster. He had, all honour to him, gone in for the veriest drudgery in his Masters' service. When the post brought the proposition of his maiden aunts, that he should provide them with some poor soul whose aching body was in need of healing country air, kindness, and good food, the Rev. Philip's only difficulty was whom to choose among the crowds around him. At last, he dispatched a hapless, half-starved mother—and her child, incurably crippled with hip-disease, the husband and father of whom was occupied "putting in his six weeks" for beating them nigh unto death. To these battered, half-alive souls, it was Heaven itself to breathe the sweet country air; their heart-wound healed over—and Margery's broths did the rest. It may as well be stated that their home and its bread-winner never saw them again. The village took care of *that*.

As for their kindly hostesses, in the rare delight of coddling the hapless waifs, they were paid, over and over again, for the sacrifice of the new black velvets. Besides—as Miss Selina discovered—with a little deft manipulation, a ribbon bow here, a lace *jabot* there, nobody at the little country dinners was any the wiser of the self-denial that brought so rich a reward.

### Lectures.

The next lecture at the Midwives' Institute and Trained Nurses' Club will be given at 12, Buckingham Street, on Friday evening, March 14th, at a quarter to eight by Dr. Tom Robinson. Subject, "Influence of the Mind upon the Body."

At the London Hospital a series of lectures on "Elementary Physiology and Medical Nursing" will be given on Wednesdays at eight p.m., beginning March 12th, by James Anderson, Esq., M.D. A limited number of ladies are admitted on payment of half-a-guinea for the course. Applications to the matron.

### The Nurses' Bookshelf.

### A NURSE AT THE FRONT.*

We are sorry to review this excellent book so late, but it has only just reached our office, probably owing to some mistake on the part of Messrs. Macmillan and Co. For some time the general public has been reading and enjoying these "Recollections," which have been favourably reviewed in all the best papers. "E. D.," or "Sister Emma," as she chooses to be called, commenced her training in 1867, but she was of an adventurous spirit and a great admirer of Dr. Livingstone; the difficulties and dangers of a nurse in England were not sufficient for her, and the first opportunity that offered she was off to Zanzibar. Sister Emma gives an amusing account of her trials at Zanzibar, which, great as they were, could not daunt her spirits. Her first case was a girl with a smashed toe, which took three months to heal; she was one of the last lot of released slaves, and knew no word of English. Constant attacks of fever took hold of all who nursed at the mission at Zanzibar, and twice Miss Jones, who was Sister Emma's colleague, thought that Sister Emma was dead. In April, 1877, Miss Jones died of typhoid, and in the following summer Sister Emma has to confess herself beaten and sail for home. She had left England in November, 1875, weighing over eleven stone. She returned in July, 1877, weighing eight stone three pounds. She took to nursing work again in London after two months' rest, and kept to it until the beginning of June, 1879. Then the Baroness Burdett Coutts gave her a chance to go out and nurse our sick and wounded soldiers in South Africa. Here she took charge of a tent at Ladysmith, and the surgical cases were sent down to her from the front. The day began at seven a.m. with getting out stores, and into the hospital by eight a.m. to prepare feeds and take temperatures. Then round with the surgeon, and then the dinners to cook and serve. The range consisted of two bricks and an iron bar, but Sister Emma, who had great faith in good feeding, got a mincing machine, and managed somehow to make savoury messes. At four p.m. those patients who wished it had tea or coffee, and at half-past six a substantial supper. Then temperatures and dressings again, and the day's work was over at nine p.m. Soon orders came to go on to Newcastle, and as no seats could be got in the post-cart, the two sisters rode the forty miles over a very rough road. Not a very comfortable reception at the end either. A mud hut for dining and sitting room, furnished with four packing cases, one for a cupboard, one for a table, and the other two for chairs; and a bell tent to sleep in, round which the soldiers had forgotten to dig a trench, so that the floor was deep in mud. All these difficulties, however, the sisters seem to have regarded as jokes, and they nursed and cleaned and cooked, and scolded the drunkards with never-failing energy. When the campaign was over, Sister Emma returned to London and took a month's rest, and then to Aix-les-Bains and elsewhere private nursing. She almost took a place offered her at Netley, but a chance of going to Antwerp turning up at the same time, the love of travel conquered even Sister Emma's great love for "the service." A few months after Sister Emma was in Kent, when she received a telegram: "Come by next train to prepare for Egypt.' During the Nile Campaign Sister Emma acted as cooking sister to the National Aid Society, and she started with good wishes from Lady Rosebery and Mrs. Gladstone. The cooking was very hot and exhausting work. During the whole time the beef-tea varied from 11 to 28 pints a day; puddings, 13 to 34; chickens, 4 to 8; fish, 2 to 5 diets; breakfast, porridge, eggs and omelettes. And there were only two sisters to do all this cooking. Sister Emma seems to consider it her chief mission to show that the best doctoring is useless where there is bad feeding. "Any nursing sister who has worked amongst the men on active service must," she says, "have had her attention drawn very often to the plates which go away almost as full as when given to the patients. When this has gone on for a week or two you will find a man has benefited little by being in hospital." "I cannot help thinking," she adds, "that if the National Aid Society would go thoroughly into the cooking branch, and perfect the work in time of peace, they would add considerably to the well-being of our sick and wounded men, and would be warmly welcomed by the Army Medical Staff." We hope all nurses who read these "Recollections" will thereby learn to pay more attention to the art of cooking, especially if they mean to travel, or to join the services. The book ends with the return from Egypt—ends as simply as it began, with few personal words, but merely with a record of facts. There is no straining after effect throughout its pages; there is no attempt at personal glorification, or at the glorification of the writer's profession. It is merely a truthful and most interesting record of a useful life, all the more admirable for the absence of all extravagant phrases.

* "The Recollections of a Nurse." By E. D. (Macmillan.)

### MEDICAL ELECTRICITY.*

This is a very condensed handbook, the letterpress being quite subsidiary to the illustrations. The latter are numerous and plain, and should be a good guide to the operator, but the book is of no use to the beginner. Unless we are mistaken, it has been written as a handy book of reference for the trained operator to carry about with him, in case he should at any time forget the exact position of any motor point. Used in this way, the book should prove valuable, especially to the many nurses who now get practical training in the use of electricity, but whose theoretical knowledge is unfortunately often slight.

* "A Handbook of Medical Electricity and Massage." By H. Newman Lawrence, A.I.E.E. (George Gill and Sons.) Price 1s.

### Examination Questions.

### PRIZE ANSWER FOR FEBRUARY.

Hæmorrhage, from the Greek word Hæmorrhagia, signifies a "flowing of blood." The various kinds subject to local treatment are arterial, venous, and bleeding from the capillaries. It is important to distinguish between arterial, venous, and capillary hæmorrhage. When blood flows out with great force and in jets, and is of a bright red colour, it is from an artery; when flowing in a steady stream and of a dark colour, it is from a vein. If from the capillaries, there is simply a gentle oozing.

Arterial hæmorrhage is infinitely more dangerous than venous, on account of the force of the current of blood being so much greater through the arteries. The principal means for arresting hæmorrhage is pressure. If the bleeding is from a vein the pressure must be applied to the limb away from the trunk; if from an artery, then between the wound and the heart—that is, above the point of bleeding.

For arterial bleeding pressure should be applied by aid of a tourniquet, or a handkerchief tied round the limb over a firm pad placed above the course of the main artery of the limb, then a stick inserted and twisted round; by this pressure is brought to bear upon the artery and the circulation stopped. If severe arterial bleeding be taking place in the neighbourhood of joints, it may be arrested by forcibly flexing the joint which is above the wound. If bleeding from a vein, a handkerchief should be tied sufficiently tight below the point of bleeding, and the limb elevated.

Bleeding from the capillaries is controlled by either direct pressure to the wounded part or by simple exposure to the cold air.

Hæmoptysis.—Coughing or spitting of blood from the lungs. Usually coughed up, and is of a bright red colour, fluid and frothy. Hæmatemesis is blood from the stomach, darker in colour than hæmoptysis, and not frothy; possibly clots mixed with food; and if it has been lying in the stomach for some time, resembles coffee grounds. Ice should be applied to the chest or pit of the stomach; or wet towels wrung out of ice cold water. Give the patient plenty of fresh cold air to breathe and ice to suck; keep quiet, and in a recumbent position. Also for hæmoptysis an inhalation of turpentine is beneficial. Epistaxis—Bleeding from the Nose.—Place the patient in an upright posture, with the head thrown back. Apply ice or cold water to the forehead, or a piece of cold metal to the back, and compress the nostrils. Give draught of iced water or lemonade. If this treatment fails, the nostrils should be plugged, an astringent being used—generally perchloride of iron steeped in lint or cotton wool.

### Appointments.

East London Nursing Society.—Miss Carter, daughter of Dr. Carter, of Wapping, has been appointed matron of the Bethnal Green Division, in place of Miss Brancher, resigned. Miss Carter trained at Shadwell, and at St. Mary's, and has lately been district nursing in Gloucester. She is used to the East-end, and is sure to be a successful worker, for her knowledge of nursing is poth practical and theoretical. We congratulate the Society on its latest matron, and we wish Miss Carter a very pleasant time at her new post.

Glamorgan and Monmouth Infirmary, Cardiff.—Miss E. Nugent, a daughter of the Archdeacon of Meath, has been appointed night sister to this infirmary. Miss Nugent trained at Addenbrookes, has been head nurse and lady superintendent to the Paulton Hospital, and subsequently head nurse, with entire charge of the male accident and surgical wards of the West Kent General Hospital, Maidstone.

Dr. T. W. Kelynack, late house physician to the Manchester Royal Infirmary, has been appointed assistant medical officer to the Manchester Workhouse Hospital at Crumpsall, and to the City Receiving and Casual Wards.

Nurses Mary Holliday and Julia C. Woldridge, of Edinburgh City Poorhouse Hospital, Craiglockhart, have been appointed head nurses of Paisley and Greenock Poorhouse Hospitals respectively.

### Notes and Queries.

### Queries.

(60) *Dispensing.*—Where can I learn dispensing and what will it cost? —*Sister Louie.*

(61) *Society of Science.*—Does such a place as the Society of Science exist? and, if so, what is its object? Are there such places as St. John's College, Penge, and Trinity College, Wallington?—*M. D.*

### Answers.

*Medical Dictionary.*—Quain's costs 30s., and is very large. Perhaps a Glossary of Anatomical, Physiological, and Biological Terms, by the late Thomas Dunman, e dited, with an Appendix, by V. H. Wyatt Wingrave, M.R.C.S. London: Griffith, Farran and Co. (2s. 6d.) would suit you.

*District Nursing.*—We beg to inform Sister Rachel that Mrs. Dacre Craven's book on "District Nursing" is published by Macmillan, price 2s. 6d.

*A Young Journeyman.*—We do not recommend physicians. Get *The Hospital Annual*, and apply to the chief of the staff of the best hospital.

*Apples and Whiskey.*—Mr. Charles Williams, Constitutional Club, Northumberland Avenue, writes us: "The late W. O'Connor, M.D., senior physician of the Royal Free Hospital, has more than once told me that the apple is a specific for intoxication from whiskey. He did not hold it to be so in the case of brandy."

*Monthly Nursing.*—Queen Charlotte's Hospital, 191, Marylebone Road, N.W., is a favourite training place for monthly nurses, who pay ten guineas for eight weeks' training and receive a certificate. At the British Lying-in Hospital, Endell-street, of which Miss Freeman is matron, about five or six monthly nurses are trained at a time for one month. Fee, £7 3s. inclusive. There are no lectures, but the nurses receive practical teaching from the matron, and nurse from thirteen to twenty cases. At Clapham Maternity Hospital, Jeffrey's Road, nurses are trained for two months for a fee of eight guineas; they have excellent lectures.

*Leather Chatelaine.*—Either from Down Brothers, 3 and 4, St. Thomas's Street, Borough, or from Krohne and Sesemann, 8, Duke Street, Manchester Square, W. *G.*—There is no such place. Your query opens up a great question into which we hope to go fully next week.

### Presentation.

F. J. Paley, M.D. Lond., on his resignation of the office of house surgeon at the Sussex County Hospital, has been presented by the hospital officers and nurses residing on the premises (joined by three gentlemen who, by their special request, were allowed to be subscribers) with a writing table and other suitable library requisites, together with a case of surgical instruments, as a mark of esteem.

### Vacancies.

The following vacancies are announced:

### Matron.

Axminster Cottage Hospital.
Bath United Hospital; £75
Essex and Colchester Hospital; £60.
Gorleston Cottage Hospital; £35.

### Nurses.

Abbey Parish New Hospital; £22.
Aimee Nursing Homes.
Burton-on-Trent Infirmary; £25.
Bristol Royal Infirmary.
Bournemouth Victoria Home; £25.
Bournemouth Nursing Institution; £25.
Brixton Institution.
Burton-on-Trent Institution; £25
Bedfordshire Institution, £23.
Bornhill Hospital, Glasgow; £24.
Church Deaconess House, Chester; £24 to £30.
Cardiff Infirmary; £24.
Cambridge Home for Nurses.
Cornelia Hospital, Poole.
Canterbury Hospital; £30.
Central London Throat Hospital; £20.
Eastbourne Sanatorium; £30.
Edinburgh City Poorhouse; £25.
Glasgow Victoria Infirmary; £20.
Glasgow Hillhead Home; £30.
General Nursing Institute.
Hampstead Home Hospital.
Hull Nurses' Home.
Jersey Institution; £25.
Kent Nursing Institution; £30.
Liverpool City Hospital; £25.
Longton Cottage Hospital.
Liverpool Institution.
Lincoln County Hospital; £20.
Middlesbrough Nursing Association.
Mr. Wilson's Institution.
Newport Institution.
North London Institution.
North West London Hospital; £24.
Nursing Institute, Isle of Wight.
Pendlebury Hospital; £20.
Royal Albert Hospital, Devonport; £25.
St. Saviour's Infirmary, Dulwich; £20.
Shoreditch Infirmary; £20.
Scarborough Institution; £25.
St. Saviour's Infirmary: £20.
St. Leonard's Nurses' Home.
St. John's Nursing Institute, Southport; £27.
Swansea and South Wales Nursing Institute; £25.
Sheffield Union; £25.
Suffolk General Hospital; £25.
Tewkesbury Hospital (night).
Weston-super-Mare Institution.
West Bromwich Hospital; £22.
Watford Union; £20.

### District Nurses.

Burton-on-Trent Institution.
Bristol Nurses' Society.
Chelsea Nursing Association.
Great Bedwyn, Wilts; £25.
Renmaemawr; £25.
South London Association.

### Probationers.

Burton-on-Trent Institution.
Bristol Royal Infirmary.
Dewsbury District Infirmary; £10.
Halifax Infirmary.
Kent Nursing Institution.
Nottingham Samaritan Hospital.
Newport and County Infirmary.
Nottingham Samaritan Hospital.
North-West London Hospital.
Nottingham Hospital for Women.
Pembrokeshire Infirmary.
Royal Surrey County Hospital.
Skin and Lock Hospital, Birmingham.
Salford Union.
Torbay Hospital.
Workhouse Nursing Association.
Weston-super-Mare Hospital.

### Amusements and Relaxation.

### FOURTH QUARTERLY WORD COMPETITION

Commenced Jan. 4th, 1890, ends March 29th, 1890.

Three prizes of 15s., 10s., 5s., will be given for the largest number of words derived from the words set for dissection.

Proper names, abbreviations, foreign words, words of less than four letters, and repetitions are barred; plurals, and past and present participles of verbs, are allowed. Nuttall's *Standard* dictionary only to be used.

N.B.—Word dissections must be sent in WEEKLY, arranged alphabetically, with correct total affixed.

The word for dissection for this, the TENTH week of the quarter, being "PRIMROSE."

| Names. | Feb. 27. | Totals. | Names. | Feb. 27. | Totals. |
|---|---|---|---|---|---|
| Holland | 62 | 705 | Nurse Clague | — | 40 |
| Red Cape | — | 132 | Lauriston | 71 | 714 |
| Nurse Marie | 53 | 499 | B. E. T. | — | 453 |
| Garfield | — | 243 | Jacko | — | 110 |
| Midget | 56 | 658 | Lady Clara | — | 258 |
| E. M. S. | — | 149 | Patience | 67 | 742 |
| Sister | — | 591 | Ecila | 65 | 730 |
| Duchess | 59 | 496 | Maude | 62 | 690 |
| Ellice | 70 | 715 | Esperance | 70 | 723 |
| Reldas | 69 | 737 | Ruby | 64 | 698 |
| Jenny Wren | 63 | 653 | M. W. | 70 | 723 |
| Mopus | — | 122 | Crystal Palace | — | 568 |
| Neptune | — | 118 | Jackanapes | 52 | 559 |
| Qu'appelle | 60 | 690 | M. M. M. | — | 25 |
| Whitehoe | 56 | 653 | A. F. | — | 318 |

### Notice to Correspondents.

N.B.—All letters referring to this page which do not arrive at 140, **Strand, London, W.C.**, by the *first post on Thursdays*, and are not addressed PRIZE EDITOR, will in future be disqualified and disregarded.

